# Effectiveness of mobile text messages v/s traditional health education on improving knowledge about oral cancer at a primary dental health care center, Cuttack, Odisha: a randomized controlled trial protocol

**DOI:** 10.3389/froh.2026.1922583

**Published:** 2026-09-14

**Authors:** Amaranath Behera, Hemamalini Rath, Utkal Kesari Mohanty, Gaurav Sharma, Saharsh Vir Jha, Krishnapriya Sekar, Anisha Dutta, Sharmistha S. Udgata, Saroj Kumar Behera, Subham Baroi, Satyaprabha Rout, Payal Maharana

**Affiliations:** Department of Public Health Dentistry, S.C.B. Dental College and Hospital, Cuttack, Odisha, India

**Keywords:** awareness, health education, health knowledge, mobile health (mHealth), mouth neoplasms, oral cancer, text messaging, WhatsApp

## Abstract

**Background:**

In developing countries like India mouth neoplasm like oral cancer shows significant and growing public health burden. It is diagnosed late due to lack in awareness which contribute to poor prognosis. Mobile health (mHealth) is effective in educating underserved rural population Health Education through Text messaging (WhatsApp) can improve knowledge at community level.

**Objective:**

This protocol evaluates the effectiveness of daily WhatsApp-based mobile text messages in improving oral cancer knowledge compared to traditional PowerPoint presentations among adult dental outpatient patients.

**Methods:**

A total of 144 eligible adults (aged 18–70 years) attending the dental OPD at CHC Bentkar will be randomized 1:1 into an intervention group (daily WhatsApp text messages) and a control group (weekly PowerPoint sessions). Oral cancer knowledge will be assessed using a validated structured questionnaire at baseline (T0), and six months (T1) post-intervention. Sample size was computed using G*Power software 3.1.9.7; Cohen's *d* = 0.5; *α* = 0.05; power = 80%) with 10% attrition allowance.

**Discussion:**

Evidence on comparative telemedicine-based approaches for oral cancer awareness in Indian primary care settings remains sparse. This trial will generate data on the scalability and durability of digital health education strategies for awareness and prevention of oral cancer, and may inform policy toward mobile-platform-delivered public health programme.

**Clinical Trial Registration:**

https://ctri.nic.in/Clinicaltrials/rmaindet.php?trialid=150355&EncHid=46498.40290&modid=1&compid=19, identifier CTRI/2026/05/111134.

## 1 Introduction

Cancer continues to impact millions of people annually and remains a serious global public health concern. According to GLOBOCAN estimates of 2022, nearly 20 million new cases of cancer and 9.7 million cancer-related deaths were recorded worldwide, of these, cancers of the lip and oral cavity were responsible for about 390,000 new cases and 188,000 mortalities, highlighting their substantial contribution to the global cancer burden. These cancers are among the most prevalent malignancies worldwide and remain an important cause of cancer-related mortality, particularly in regions where tobacco and areca nut use are prevalent (1). While oral cancer is a global health concern, its burden is not evenly distributed. The majority of cases and deaths occur in Asia, particularly in South-Central Asia, where tobacco and areca nut consumption are common. India bears a disproportionately large share of this burden, with oral cancer ranking one of the most prevalent cancers and a major contributor to cancer-related death (2). This scenario is due to the lack of knowledge and awareness of the common people regarding oral cancer and their heavy use of alcohol, tobacco, betel quid chewing, and areca nut products (3).

Patients’ knowledge regarding oral squamous cell carcinoma (OSCC) is crucial for early detection and primary prevention, both of which have a direct impact on survival rates and prognosis. Patient delay occurs when people fail to detect early signs of OSCC, which are frequently painless in their early stage (4). Human behavior provides avenues for focused intervention and contains the key to comprehending the etiology of cancer. The impact of cancer can be minimized by effective interventions to lower modifiable risk factors, early case diagnosis, and proper management and care for those who already have the disease (5).

Health education is the core instrument for empowering patients well-being by enhancing their knowledge, motivation, and self-management skills. Educational interventions grounded in behavioral change theories, such as the social cognitive theory, are directly linked theoretical knowledge to preventive actions (6). The traditional method of imparting health education has been effective in improving the awareness of oral cancer. Healthcare education is continuously changing with the advent of the digital era, and teaching strategies that incorporate digital presentations are currently receiving greater attention (7). Based on the burden of oral diseases, the WHO Global Oral Health Programme, as part of the joint WHO–ITU Be Healthy Be Mobile (BHBM) initiative, has made the development of the m Oral Health programme one of its priorities (8). WhatsApp, one of the smart phone apps, has become a potent communication tool due to its high adoption rate (9), familiarity, and low cost. Teledentistry is providing on-demand educational reinforcements like inspirational text messages.This ongoing involvement makes it easier to include customized oral health promotion into patients’ everyday schedules (10).

Existing literature comparing traditional and digital approaches suggests that while traditional education establishes foundational knowledge, digital approaches excel in sustaining long-term compliance and improving patient attitudes (11). Despite the growing body of mHealth research, significant gaps remain in the existing literature. Digital research often focuses on urban populations. Furthermore, while evidence supports WhatsApp-mediated health education, robust data regarding its efficacy for the long-term retention of oral cancer knowledge in primary healthcare settings, particularly in India, remains sparse. Therefore, this study measures the effectiveness of mobile text messages (WhatsApp) in improving knowledge of oral cancer among patients visiting primary dental health care centers, and thereby promoting oral cancer prevention and control in this country. We hypothesize that Mobile text message-based health education is more effective than traditional health education in improving and sustaining knowledge regarding oral cancer among patients attending a primary dental health care center.

### 1.1 Aim

To measure the effectiveness of mobile text messages i.e., WhatsApp, as compared to traditional health education via PowerPoint presentation to improve the knowledge of oral cancer among dental OPD patients in primary dental health care center, Cuttack, Odisha.

### 1.2 Objective

To assess the change in knowledge score regarding oral cancer through daily mobile text messages via WhatsApp as compared to the traditional health education via PowerPoint presentation.

## 2 Methods and analysis

The study duration spans from July 2026 to June 2027. Ethical clearance was obtained from the Institutional Ethics Committee (IEC), S.C.B. Dental College and Hospital, Cuttack, Odisha (Reg. No. ECR/1355/Inst./OD/2020), this is prospectively registered with the Clinical Trials Registry of India (CTRI/2026/05/111134).

### 2.1 Study design

This study is a two-arm, parallel-group randomized controlled trial (RCT), with 1:1 allocation ratio designed to recruit adult dental OPD patients attending CHC Bentkar (a primary dental health care center), Cuttack District, Odisha, India, based on their willingness to participate.

### 2.2 Study population and eligibility criteria

#### 2.2.1 Inclusion criteria

- Adult patients attending the dental OPD at CHC Bentkar, Cuttack.
- Age between 18 and 70 years.
- Ownership of a smart phone with an active WhatsApp account.
- Able to read and write local language(odia)

#### 2.2.2 Exclusion criteria

- Patients who decline to provide informed consent.
- Individuals already enrolled in another text message-based intervention study.
- Patients with a prior personal or first-degree family history of oral cancer diagnosis.

### 2.3 Sample size calculation

An independent samples t-test (two-tailed) comparing the difference between two independent means was used in G*Power software (version 3.1.9.7) (12). With an alpha error probability of 0.05, a moderate effect size (Cohen's *d* = 0.5) (13, 14) and a statistical power of 80% with an allocation ratio of 1:1, the calculated minimum required sample size was 64 participants per group (total *n* = 128). Considering for an anticipated 10% dropout rate, the calculated final sample size was 144 participants (72 per group).

### 2.4 Questionnaire

Data will be collected using a self-administered, validated, close-ended structured questionnaire developed by the investigators based on previous literatures (15–18). The questionnaire consists of two sections. The first section includes questions on sociodemographic characteristics and history of tobacco use. The second section assesses awareness of oral cancer using 36 items categorized into four domains: clinical signs and symptoms (10 items), risk factors (9 items), prevention (7 items), and available treatment options (10 items). Some items are negatively worded to minimize acquiescence response bias. The questionnaire is translated to local language (Odia) by linguistic experts before administration.

The English version was forward-translated into Odia by two independent bilingual translators, and the reconciled Odia version was back-translated into English by a third translator who had not seen the original. Discrepancies between the back-translation and the source were reviewed by the expert committee and resolved by consensus, with attention to conceptual rather than literal equivalence and to the local terminology used for tobacco and areca nut products. The final Odia version was pre-tested for comprehension in 32 dental outpatients who will not be included in the trial.

Face validity was assessed by a panel of experts to ensure the clarity and relevance of the questionnaire. Content validity was evaluated by seven subject experts from four specialties: Oral and Maxillofacial Surgery, Oral Pathology and Microbiology, Oral Medicine and Radiology, Public Health Dentistry, and Head and Neck Onco-surgery. The scale-level Content Validity Index (S-CVI) was 0.80. The reliability of the questionnaire was assessed using internal consistency (Cronbach's alpha = 0.87) and test-retest reliability (correlation coefficient = 0.74).

Responses will be recorded using a three-response option format (agree, disagree, and don't know). For analysis, responses will be dichotomized, with correct answers scored as 1 and incorrect or “don't know” responses scored as 0. The overall awareness score will be calculated by summing the scores of all 36 items, with a possible total score ranging from 0 to 36, where higher scores indicate greater awareness of oral cancer.

### 2.5 Data collection consent

A written informed consent form will be provided to and obtained from all participants prior to enrolment. Study objectives, procedures will be explained by principal investigator and participation will be entirely voluntary; participants may withdraw at any time without consequences to their clinical care. All data will be anonymised and consent will cover use of participant data for study purposes. The consent form will state explicitly that participants allocated to the intervention arm will be invited to join a WhatsApp group in which their mobile number will be visible to other group members, and that they may decline group participation without affecting any other aspect of their involvement in the trial.

### 2.6 Randomization and allocation concealment

Eligible participants will be identified by the principal investigator during routine dental OPD visits at CHC Bentkar. Recruitment will proceed through consecutive sampling of willing participants until the target sample size is achieved.

Participants will be randomly allocated to either the intervention group or the control group, following a 1:1 allocation ratio, using a computer-generated random sequence (http://www.randomization.com) created by an independent researcher (K.S.) who is not involved in participant recruitment, intervention delivery, or outcome assessment. Allocation concealment will be achieved using sequentially numbered, opaque, sealed envelopes (SNOSE) prepared by another independent researcher (S.V.J.). After obtaining written informed consent and completing the baseline examination, the principal investigator (A.B.) will open the next sequentially numbered envelope to reveal the participant's group allocation. Investigator responsible for recruitment and enrolment had no prior knowledge of upcoming sequence. The envelopes will be identical, opaque, sealed, and opened strictly in sequence to ensure that the allocation remains concealed until assignment, thereby minimizing selection bias.

Due to the nature of the intervention, blinding of principal investigator and study participants is not possible for this trial. However, the outcome assessor (H.R.) will remain blinded to group allocation to minimize assessment bias.

### 2.7 Interventional methods

The questionnaire will be administered in a private area of the dental OPD by the principal investigator, assisted by a trained investigator who is not involved in intervention delivery. Baseline (T0) data will be collected after consent and before allocation, so that the assessor is unaware of group assignment at that point. Completion is expected to take approximately 5–8 minutes. Follow-up (T1) data will be collected six months after completion of the intervention in the participant's own arm; participants will be contacted by telephone to schedule the follow-up visit, and up to three contact attempts will be made before a participant is recorded as lost to follow-up.

Eligible participants will be recruited based on the randomization either to the intervention group (daily oral cancer-specific Whats App messages) or the control group (traditional health education via PowerPoint presentation).

#### 2.7.1 Intervention group: WhatsApp text messages

Participants in the experimental group will receive validated 42 messages, oral cancer-specific daily for six week. Messages include text and pictures relevant to oral cancer awareness. Message composition (two to four sentences in Odia plus a clinical photograph) validated through expert review for accuracy and readability; comprehension pilot testing is same as in power point presentation. Educational messages will be sent one-to-one from a dedicated study mobile number held by the principal investigator, so that participants’ numbers are not disclosed to one another during message delivery. The weekly discussion takes place in a closed WhatsApp group in which member numbers are visible to other members; this is disclosed in the consent form, participation in the group is optional, and participants who decline may submit queries individually to the study number and receive the same clarification. The group will be administrator-restricted, will contain no participant identifiers other than the mobile number, and will be deleted at the end of the intervention period. The principal investigator will monitor the delivery status of every sent message; undelivered messages will be resent, and participants with persistent delivery failures will be contacted by telephone. To prevent contamination between groups, participants will be instructed not to forward any messages outside the group.

#### 2.7.2 Control group: traditional health education via PowerPoint presentations

Participants in the control group will receive health education through weekly PowerPoint presentations at CHC Bentkar, one session per week for one month. Each session duration of 40 minutes will include internal structure 10 minutes of previous lecture discussion, 15-minute standardised presentation from a fixed slide set, 10 minutes of question-and-answer and group discussion. Session topics are as follows:

1. Session 1: Diagnosis, symptoms and complications of oral cancer
2. Session 2: Prevalence of oral cancer and its risk factors— smoking, smokeless tobacco, and alcohol consumption.
3. Session 3: Prevention behavior —Tobacco cessation, appropriate diet, and dental hygiene.
4. Session 4: Benefits of prevention behavior; review of previous sessions; training in oral self-examination (OSE) to detect premalignant lesions.

The focus of the intervention content for both groups will cover: tobacco types, clinical signs and symptoms of oral cancers as well as precancerous conditions; Both groups will be trained to perform oral self-examination to identify potentially malignant or premalignant lesions in themselves or family members, and to refer such cases to S.C.B. Dental College and Hospital (SCB DCH), Cuttack.

### 2.8 Outcome measures

The primary outcome will be the change in mean knowledge score from baseline (T0) to six months post-intervention (T1), compared between the intervention and control groups.

The secondary outcome will be the change in mean knowledge score at six months post-intervention (T1) within groups.

### 2.9 Harms

No significant harms are anticipated from participation in either educational intervention. Adverse events will be monitored through passive surveillance, documented, assessed for causality, and reported to the IEC.

### 2.10 Participant timeline

Participants will be assessed at two time points: baseline (T0) and six months post-intervention (T1). The schedule of enrolment, intervention delivery, and assessments is summarised in the flow diagrams embedded in this protocol (Figures 1, 2).

**Figure 1:**
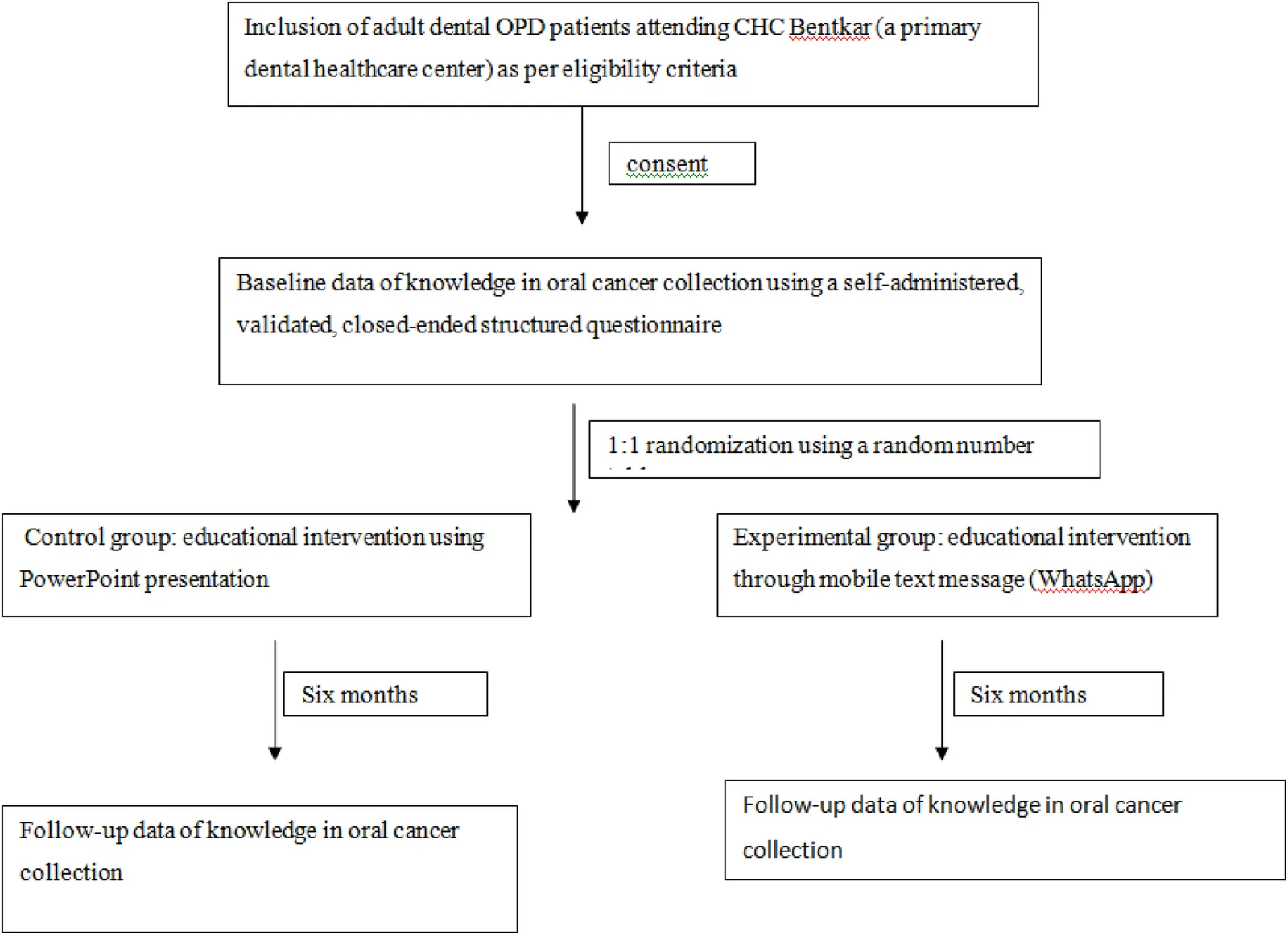
Flow diagram for the randomized controlled trial comparing WhatsApp-based oral cancer education with traditional PowerPoint-based health education among dental OPD patients at CHC Bentkar, Cuttack, Odisha, India.

**Figure 2:**
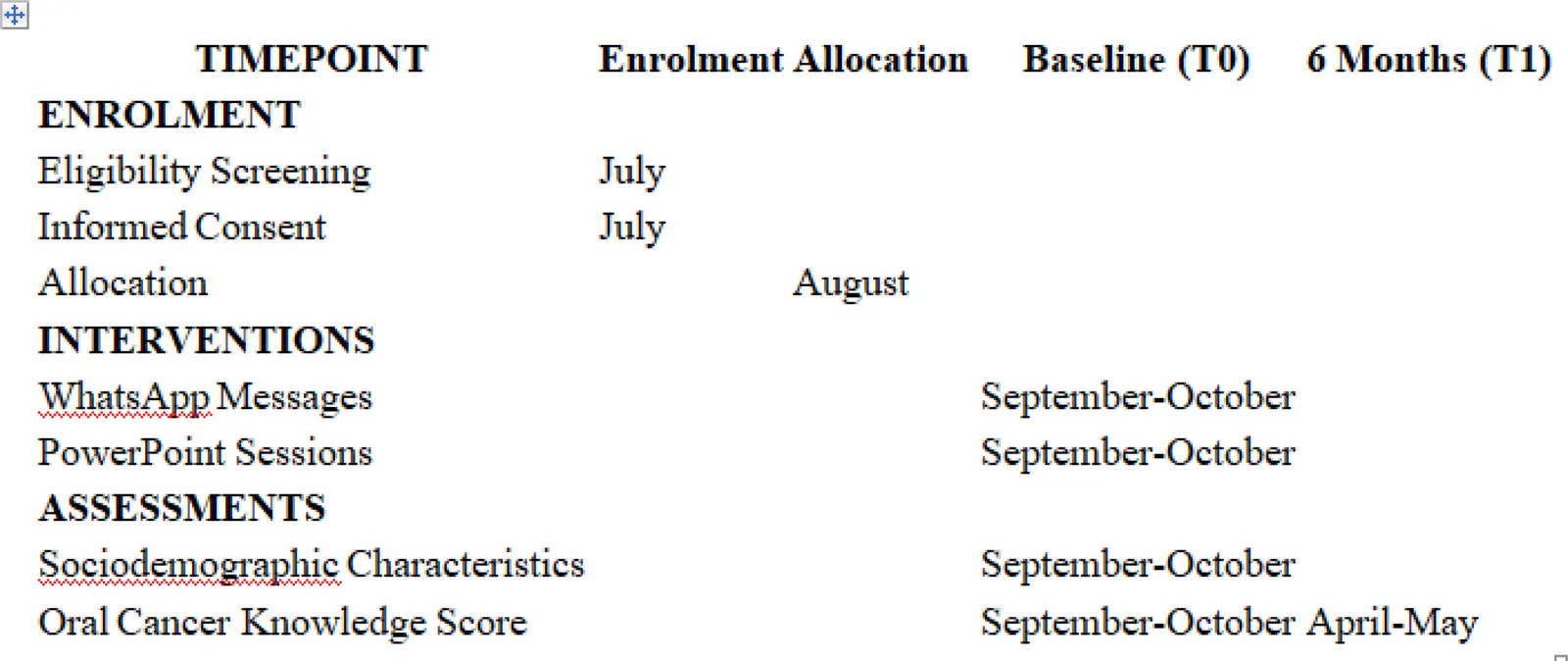
Spirit schedule of enrolment, interventions, and assessments for the randomized controlled trial comparing WhatsApp-based oral cancer education with traditional PowerPoint-based health education among dental OPD patients at CHC Bentkar, Cuttack, Odisha, India.

### 2.11 Data management

All collected data will be coded and entered into a password-protected electronic database accessible only to the research team. Data accuracy will be verified through double-entry checking and periodic review. Participant identifiers will be stored separately from study data.

### 2.12 Protocol amendments

Any substantive amendments will be communicated to the CTRI registry and IEC.

### 2.13 Ancillary and post-trial care

Participants identified with suspicious oral lesions will be promptly referred to SCB DCH, Cuttack, for clinical evaluation. No ancillary interventions are planned.

### 2.14 Flow diagram of methodology

The overall methodology of the trial is summarised in two figures. Figure 1 presents the CONSORT-style flow diagram, showing the sequence from inclusion of adult dental OPD patients attending CHC Bentkar as per the eligibility criteria, through written informed consent, baseline (T0) assessment of oral cancer knowledge using the self-administered, validated, closed-ended structured questionnaire, and 1:1 randomization using a computer-generated random sequence, to allocation into the control group (educational intervention using PowerPoint presentation) and the experimental group (educational intervention through mobile text messages via WhatsApp), with follow-up (T1) data on oral cancer knowledge collected in both arms six months after completion of the intervention. Figure 2 presents the SPIRIT schedule of enrolment, interventions and assessments, indicating the timing of eligibility screening and informed consent (July), allocation (August), delivery of the WhatsApp and PowerPoint interventions together with baseline sociodemographic and knowledge assessment (September–October), and the six-month oral cancer knowledge assessment (April–May).

## 3 Ethics and dissemination

Ethical clearance has been obtained from the Institutional Ethics Committee, S.C.B. Dental College and Hospital, Cuttack, Odisha (Reg. No. ECR/1355/Inst./OD/2020). Written informed consent will be obtained from all participants prior to enrolment. The trial was registered with the Clinical Trials Registry India (CTRI) prior to commencement of recruitment (CTRI/2026/05/111134).

Outcomes will also be communicated to study participants and pertinent health authorities in Odisha.

## 4 Statistical analysis

Statistical analysis will be performed using IBM SPSS Statistics, Version 26.0 (IBM Corp., Released 2019. Armonk, NY: IBM Corp.). Continuous variables will be reported as mean ± standard deviation (SD) or median (interquartile range, IQR), and categorical variables as frequencies and percentages. Baseline comparability between groups will be tested using the independent samples t-test for continuous variables and the chi-square test for categorical variables.

### 4.1 Primary analysis

The primary analysis will be an analysis of covariance (ANCOVA) comparing the mean knowledge score at six months (T1) between the intervention and control groups, with the baseline (T0) score as covariate and study group as fixed factor. ANCOVA was preferred to a t-test on change scores because adjustment for baseline increases precision.

### 4.2 Secondary analyses

Within-group change from T0 to T1 will be tested by paired samples t-test, or the Wilcoxon signed-rank test where indicated. Cohen's d and partial eta squared will be reported alongside p values, with 95% confidence intervals for all between-group differences. All tests will be two-tailed at *p* < 0.05.

### 4.3 Missing data

The extent and pattern of missing outcome data will be compared between groups and handled by multiple imputation by chained equations under a missing-at-random assumption.

## 5 Discussion

This paper presents the protocol for a RCT evaluating the comparative effectiveness of two modes of health education for improving oral cancer awareness and knowledge in adult dental OPD patients attending a primary dental health care center (CHC i.e Community Health Center) in Odisha state of India. The study can address a well-defined evidence gap in the mHealth literature for oral cancer.

Oral cancer carries a disproportionate burden in India, and knowledge deficits at the community level are associated with delayed help-seeking and late-stage presentation (19, 20). While health education has long been recognized as a core strategy in prevention, the rapid growth of mobile technology offers a paradigm shift in how such education can be delivered, particularly to geographically underserved populations (5, 8). Primary health care centers such as CHC Bentkar serve as critical contact points between the health system and the community, and interventions delivered at this level reach the population that carries the greatest share of risk.

A systematic review by Ahuja et al. on interventions to improve oral cancer knowledge identified 27 eligible studies and concluded that research should prioritise testing feasibility, long-term effectiveness and sustainability, especially in low- and middle-income countries. Notably, only two of the reviewed studies used WhatsApp as a delivery platform, underscoring the novelty and relevance of the present trial (20).

The evidence base supporting WhatsApp-mediated health education is steadily growing. Nayak et al. reported a highly significant improvement in oral cancer knowledge scores in the digitally educated group compared with a traditional PowerPoint group (t = −15.05, *p* ≤ 0.001) (9). Jeihooni and Harsini similarly demonstrated that an educational intervention supplemented by WhatsApp messaging significantly improved oral cancer prevention behaviours and reduced nicotine dependence among hookah users at six months of follow-up (21). The present trial extends this evidence to an adult primary care population in an Indian setting, which is more representative of those carrying the highest burden of oral cancer risk.

The six-month follow-up is a design strength. Most existing mHealth trials report outcomes at one month or less, leaving knowledge retention largely unanswered (20). Measuring knowledge six months after the intervention has ended will show whether any gain persists once message delivery stops, and will inform decisions about booster or re-intervention strategies.

Several limitations merit acknowledgement. The two arms differ in both duration and frequency of exposure: the intervention group receives daily messages over six weeks, whereas the control group attends four weekly sessions over four weeks. The comparison is therefore between two educational packages as each would realistically be delivered, not a dose-matched comparison of media, and a difference in knowledge score cannot be attributed to mode of delivery alone. To keep the follow-up interval equal, the six-month assessment will be timed from completion of the intervention in each arm rather than from randomization. The open-label design, while unavoidable, may introduce performance bias, although blinded outcome assessment limits detection bias. Requiring an active smartphone with WhatsApp introduces a selection bias more consequential than penetration figures suggest: it excludes the least digitally connected patients, who are frequently the least literate and most disadvantaged and may carry the highest risk. The single-centre setting may limit generalisability, although it allows rigorous implementation and quality control. Social desirability may influence self-reported knowledge scores.

### 5.1 Future recommendations

If daily WhatsApp messaging is found to improve and sustain oral cancer knowledge, several lines of further work follow. The comparison should be repeated in a multicentre design across districts with differing levels of digital access, to establish whether the effect holds outside a single community health centre. Subsequent trials should extend the outcome set beyond knowledge to preventive behaviour, uptake of oral self-examination and referral completion.

## 6 Conclusion

This RCT protocol describes a methodologically robust study to determine whether daily mobile text messages via Whats App can more effectively improve oral cancer knowledge among adult dental OPD patients than traditional health education via PowerPoint presentations at a primary health care center in Cuttack, Odisha. Findings from this trial will contribute meaningful evidence on the potential of mHealth-based approaches for oral cancer prevention in high-burden settings and may inform policy toward scalable digital health education strategies in India.
