# Supplementary material for: Effectiveness of mobile text messages v/s traditional health education on improving knowledge about oral cancer at a primary dental health care center, Cuttack, Odisha: a randomized controlled trial protocol
Source: Front Oral Health. 2026 Sep 14;7:1922583. doi: 10.3389/froh.2026.1922583 (PMC13616792; doi:10.3389/froh.2026.1922583)
Supplement: Supplementary file 1 [file Datasheet1.pdf]

ନିମ୍ନ ଲିଖିତ ପ୍ରଶ୍ନ ଗୁଡିକ ମୁଖ ଗହ୍ୱର ରୋଗ ସମ୍ବନ୍ଧେ ଆପଣଙ୍କର ଜ୍ଞାନ ସହିତ ଜଡିତ | ଦୟା କରି ପ୍ରତ୍ୟେକ ପ୍ରଶ୍ନ ଗୁଡିକୁ ଭଲ ଭାବରେ ପଢନ୍ତୁ ଏବଂ ନିମ୍ନ ଦିଆଯାଇଥିବା ବିକଳ୍ପ ଗୁଡିକ ମଧ୍ୟରୁ ଗୋଟିଏ କୁ ଠିକ୍ କରନ୍ତୁ |

Name- ______________________ Age- _____ Gender- _____Education- ______________

WhatsApp no - ________________ occupation- ___________Income(monthly)-__________

S.no	Question	ସମ୍ପୂର୍ଣ୍ଣ ରାଜି	ଜଣା ନାହିଁ	ସମ୍ପୂର୍ଣ୍ଣ ଅରାଜି
1	ପାଟି ରେ ଧଳା/ନାଲି ଦାଗ ମୁଖ ଗହ୍ୱର କ୍ୟାନସର ର ପ୍ରଥମ ଲକ୍ଷଣ ହୋଇପାରେ			
2	ପାଟିର କୌଣସି ଘା ଦୁଇ ସପ୍ତାହ ପର୍ଯ୍ୟନ୍ତ ନ ଶୁଖିଲେ ଡାକ୍ତର ଙ୍କୁ ଦେଖାଇବା ଦରକାର			
3	ପାଟିରେ କୌଣସି ସ୍ଥାନ ରେ ମାଂସ ବୃଦ୍ଧି ହେବା ବିପଦ ଜନକ ହୋଇପାରେ			
4	ପାଟିର କୌଣସି ଅଂଶ ହଠାତ ବଧିରା ଲାଗିଲେ କ୍ୟାନସର ଜନିତ ହୋଇପାରେ			
5	ପାଟି ଜଳା ପୋଡ଼ା ହେବା କ୍ୟାନସର ର ଏକ ମାତ୍ର ଲକ୍ଷଣ ଅଟେ			
6	ବହୁତ ଦିନ ଧରି ପାଟି ଖୋଲିବାରେ ଅସୁବିଧା ହେବା ମୁଖ ଗହ୍ୱର କ୍ୟାନସର ର ପୂର୍ବ ସ୍ଥିତି ହୋଇପାରେ			
7	ବହୁତ ଦିନ ପର୍ଯ୍ୟନ୍ତ ଖାଦ୍ୟ ଢୋକିବାରେ ଅସୁବିଧା କ୍ୟାନସର ର ଲକ୍ଷଣ ହୋଇପାରେ			
8	କିଛି କାରଣ ନଥାଇ ସ୍ୱର ରେ ହଠାତ ଅସ୍ୱାଭାବିକ ପରିବର୍ତ୍ତନ ହେଲେ ଡାକ୍ତର ଙ୍କ ପରାମର୍ଶ କରିବା ଉଚିତ୍			
9	ମୁଖ ଗହ୍ୱର କ୍ୟାନସର କେବଳ ଜିଭ ରେ ହୋଇଥାଏ			
10	ମୁଖ ଗହ୍ୱର କ୍ୟାନସର ର ବେକ କୁ ବ୍ୟାପି ଯିବାର ସମ୍ଭାବନା ବହୁତ ଅଧିକ ଅଟେ			
11	ଅତ୍ୟଧିକ ମିଠା ସେବନ କରିବା ଦ୍ୱାରା କ୍ୟାନସର ହେବାର ସମ୍ଭାବନା ଥାଏ-			
12	ଦାନ୍ତ ଓ ପାଟି କୁ ଭଲଭାବରେ ସଫା ନ କରିବା କ୍ୟାନସର ଏକ ମାତ୍ର କାରଣ			
13	ବୟସ ବୃଦ୍ଧି ହେବା ସହ କ୍ୟାନସର ହେବାର ସମ୍ଭାବନା ବୃଦ୍ଧି ପାଇଥାଏ			
14	ବିଶ୍ୱ ରେ ତମାଖୁ ସେବନ ମୁଖ ଗହ୍ୱର କ୍ୟାନସର ର ପ୍ରମୁଖ କାରଣ ଅଟେ			
15	ମଦ୍ୟପାନ ସହିତ ଧୂମ୍ରପାନ ଥିଲେ ତାହା ମୁଖ ଗହ୍ୱର କ୍ୟାନସର ସମ୍ଭାବନା କୁ ବଢାଇ ଦିଏ			
16	ମୁଖ ଗହ୍ୱର କ୍ୟାନସର ଏକ ଛିଆଁ ରୋଗ ଅଟେ			
17	ମୁଖ ଗହ୍ୱର କ୍ୟାନସର ର ସମପରିବାର ର ବ୍ୟକ୍ତି ମାନଙ୍କୁ ବେଶୀ ପ୍ରଭାବିତ କରିଥାଏ			
18	କେବଳ ଭାଗ୍ୟ ଦୋଷରୁ ହିଁ କ୍ୟାନସର ହୋଇଥାଏ			
19	ମୁଖ ଗହ୍ୱର କ୍ୟାନସର ଏକ ଜୀବାଣୁ ଜନିତ ରୋଗ			
20	ତୁରନ୍ତ ତମାଖୁ ବର୍ଜନ ଦ୍ୱାରା ମୁଖ ଗହ୍ୱର କ୍ୟାନସର ର ସମ୍ଭାବନା କୁ ବହୁତ ମାତ୍ରା ରେ ଏଡାଇ ଦିଆଯାଇପାରେ			

21	ପାଟିରେ ଧଳା କିମ୍ବା ନାଲି ଦାଗ ଦେଖାଯିବା ମାତ୍ରେ ଦାନ୍ତ ଡାକ୍ତର ଙ୍କୁ ପରାମର୍ଶ କରିବା ଉଚିତ୍			
22	ବିଡି/ସିଗାରେଟ ଧୂଆଁ ର ସଂସ୍ପର୍ଶ ରେ ଆସନ୍ତୁ ନାହିଁ			
23	ଭଲ ଭାବରେ ଖାପ ନ ଖାଉଥିବା କୃତ୍ରିମ ଦାନ୍ତ କୁ ଯଥା ଶୀଘ୍ର ବଦଳାଇ ଦବା ଉଚିତ୍			
24	ଭଙ୍ଗା କିମ୍ବା ଧାରୁଆ ଦାନ୍ତ କୁ ଯଥା ଶୀଘ୍ର ଉପାଡି ଦେବା ଦ୍ୱାରା କ୍ୟାନସର ହେବାର ସମ୍ଭାବନା କମିଯାଏ			
25	ମୁଖ ଗହ୍ୱର କ୍ୟାନସର ର ବ୍ୟକ୍ତି ର ଲାଳ ର ସଂସ୍ପର୍ଶରେ ଆସିବା ଅନୁଚିତ୍			
26	ମୁଖ ଗହ୍ୱର କ୍ୟାନସର ର ଚିକିତ୍ସା ସମ୍ଭବ ନୁହେଁ			
27	ମୁଖ ଗହ୍ୱର କ୍ୟାନସର ଥିବା ବ୍ୟକ୍ତି ଯଥା ଶୀଘ୍ର ମୃତ୍ୟୁ ବରଣ କରନ୍ତି			
28	ଅସ୍ତ୍ରୋପଚାର ଦ୍ୱାରା ମୁଖ ଗହ୍ୱର କ୍ୟାନସର କୁ ସମ୍ପୂର୍ଣ୍ଣ ମାତ୍ର ରେ ଠିକ୍ କର ଯାଇପାରେ			
29	ମୁଖ ଗହ୍ୱର କ୍ୟାନସର ଜନିତ କୌଣସି ଲକ୍ଷଣ ଦେଖା ଗଲେ ଦାନ୍ତ ଡାକ୍ତର ଙ୍କୁ ହିଁ ପରାମର୍ଶ କରିବା ଉଚିତ୍			
30	ପ୍ରାଥମିକ ସ୍ତରରେ ଧରା ପଡିଗଲେ ଚିକିତ୍ସା ରୁ ବିଶେଷ ଲାଭ ମିଳିଥାଏ			
31	କେମୋଥେରାପି ମୁଖ ଗହ୍ୱର କ୍ୟାନସର ର ଏକ ମାତ୍ର ଚିକିତ୍ସା ପ୍ରଣାଳୀ ଅଟେ			
32	ହୋମିଓପ୍ୟାଥି/ ଆୟୁର୍ବେଦିକ ଚିକିତ୍ସା ଦ୍ୱାରା କ୍ୟାନସର କୁ ସମ୍ପୂର୍ଣ୍ଣ ମାତ୍ରାରେ ଠିକ୍ କରାଯାଇପାରେ			
33	ବାୟୋପ୍ସି/ ମାଂସ ପରୀକ୍ଷା କରିବା ଦ୍ୱାରା କ୍ୟାନସର ଚାରି ଆଡକୁ ବ୍ୟାପି ଯିବାର ସମ୍ଭାବନା ବଢିଯାଏ			
34	ତମାଖୁ ସେବନ ବର୍ଜନ କରିବା ଦ୍ୱାରା କ୍ୟାନସର ର ଆକାର ହ୍ରାସ ପାଇଥାଏ			
35	ଗୁଣି ଗାରଡ଼ି କିମ୍ବା ଝଡ଼ା ଫୁଙ୍କା ଦ୍ୱାରା କ୍ୟାନସରକୁ ଭଲ କରାଯାଇ ପାରେ			
36	ବାୟୋପ୍ସି/ ମାଂସ ପରୀକ୍ଷା ଦ୍ୱାରା ମୁଖଗହ୍ୱର କ୍ୟାନସର ର ପ୍ରକାର ନିର୍ଣ୍ଣୟ କରାଯାଇପାରେ			
